# Definition of NPSLE: Does the ACR Nomenclature Still Hold?

**DOI:** 10.3389/fmed.2018.00138

**Published:** 2018-05-31

**Authors:** Jessica Fernandes Vivaldo, Jaqueline Cristina de Amorim, Paulo Rogério Julio, Rodrigo Joel de Oliveira, Simone Appenzeller

**Affiliations:** ^1^Autoimmune Lab, School of Medical Science, University of Campinas, Campinas, Brazil; ^2^Graduate Program in Child and Adolescent Health, School of Medical Science, University of Campinas, Campinas, Brazil; ^3^Rheumatology Unit, School of Medical Science, University of Campinas, Campinas, Brazil

**Keywords:** neuropsychiatric SLE, ACR criteria, systemic lupus erythematosus (SLE), cognition disorders, criteria

## Abstract

Systemic lupus erythematosus (SLE) patients have frequently neuropsychiatric manifestations. From the first description of coma in 1875, a variety of manifestations has been described to occur in SLE. However, the lack of standardization reduced the comparability of published studies. In 1999, the American College of Rheumatology published guidelines to define neuropsychiatric nomenclature in SLE. This was the first step toward uniform diagnostic criteria. Several studies have been published since then applying the ACR criteria and frequencies of different manifestations can now be compared between cohorts. Although these criteria are diagnostic, therapeutic approach to different manifestations varies according to nature and severity of the manifestations. Herby, we will review the different definition for NPSLE published, and determine advantages and limitation.

## Introduction

Systemic lupus erythematosus (SLE) is a chronic, multisystem, autoimmune disease (1–3). Depending on the type of manifestations included and the method used for evaluation, the frequency of neuropsychiatric (NP) manifestations varies widely across studies (3, 4). NP involvement may be considered primary if result directly from immune-mediated injury or secondary when related to treatment, infections, metabolic abnormalities, or other systemic manifestations, not related to SLE (3, 5). In major cohorts, approximately 60% of NP manifestations are due to secondary causes (4–8).

The diagnosis of primary NP involvement is often difficult, as both focal and diffuse manifestations may occur; and there is no gold standard for diagnosis. A high index of clinical suspicion is necessary, in addition to laboratory and neuroimaging findings, to support diagnosis, and exclude confounding diagnosis (1–5).

The pathogenesis of primary NPSLE is yet not completely elucidated, and considering the diversity of manifestations, probably one or more mechanism of pathogenicity is implicated (3, 5). Several studies have described autoantibodies and cytokines as possible mediators, affecting cerebral vasculature, and/or interfering with neuronal connectivity (3, 5, 6).

From the first description of coma in the Nineteenth century, a growing number of NP manifestations have been attributed to SLE. However, the lack of standardization reduced the comparability between published studies (9–11). Over the last decades, several protocols have attempted to overcome this issue. In this article, we will review the different criteria published and discuss advantages and limitations.

## Toward standardization of NPSLE. historical overview

The NP manifestations are generally diverse in symptomatology; extend of cerebral involvement and disease severity (1–7). Several studies have shown that the presence of NPSLE, independent of its etiology, is associated with greater morbidity and mortality (12–17). Several authors have tried to develop classification criteria to enhance patient care and facilitate clinical and basic science research (11). From the initial studies in NPSLE, the need for differentiating primary from secondary NPSLE has become evident (18–21).

Kassan and Lokshin proposed one of the first classification criteria in 1979 (22). They defined a CNS event as “an abnormality of neurologic function, identified by reliable history or examination, and noted as a change from a prior state.” To consider it as an event related to SLE, infection, trauma, malignancy, and other independent neurologic diseases had to be excluded (22). Eight clinical manifestations were listed, however not defined: seizure, disturbance of consciousness, disturbance of mental function, neuropathy, motor disorder, movement disorder, meningitis, and encephalitis (22). Each event was additionally codified into presence or absence of systemic disease, laboratory abnormalities, function and chronology (22). The authors recognized that the coding system had its challenge; however, simple qualitative or descriptive statement would not adequately describe the current physiopathology knowledge (22).

Although Kassan and Lokshin made a first step toward some needed classification criteria, limitations of these criteria included the lack of definition of individual clinical manifestations and absence of validation in independent cohorts (Table 1).

**Table 1:** Compression of different Neuropsychiatric (NP) criteria.

	**Kassan and Lockshin [22]**	**How et al [24]**	**1999 NP ACR criteria [28, 29]**
NP manifestations included	Seizure Disturbance of consciousness Disturbance of mental function Neuropathy Motor disorder Movement disorder Meningitis Encephalitis	Major symptoms (seizures, focal motor or sensory deficits, generalized disturbances, psychosis and organic brain syndrome) Minor symptoms (paresthesia without objective findings, clumsiness without objective findings, persistent headache, pseudopapilledema, benign intracranial hypertension, reactive depression, mood swings, cognitive disorders, severe anxiety and behavioral problems)	12 CNS and 7 PNS (Table 2)
Definition of NP manifestations	No	Not given	Yes
Exclusion criteria	Infection,Trauma, neoplasm, and other independent neurologicdiseases	Not given	Individually listed for each of the 19 NP manifestations
Additional information	Codification: Presence/absence of systemic disease activity; laboratory abnormalities; impairment of function and chronology	Diagnosis of NPSLE: 1 major criterion; or 1 minor criterion plus abnormality shown on electroencephalogram, brain scan,CSF examination or cerebral angiogramFurther classified in focal and diffuse	Comprehensive investigation protocol for each individual manifestation
Advantage	Inclusion of function impairment and chronology	Translational study exploring pathogenesis	Comprehensive definitionValidated in different cohorts
Limitations	No definitions of individual manifestationsNo validation	Based on chart reviewNo validation in other cohorts or studies	Lack of attribution; chronology

*CNS, central nervous system; NP, neuropsychiatric; PNS, peripheral nervous system*.

In the following years, the American College of Rheumatology (ACR) classification criteria for SLE were developed (23). In the neurological domain, only seizures and psychosis were included. In dividual definitions of individual neurological criteria were not given, however, exclusion criteria were defined (23).

Due to the lack of established criteria, several researchers developed independent criteria to be used in basic or translational studies (24–27). In order to study the role of antineuronal antibodies in NPSLE, How et al classified SLE patients according to major and minor NP symptoms (24). The following manifestations were considered major symptoms: seizures, focal motor or sensory deficits, generalized disturbances, psychosis, and organic brain syndrome (24). Manifestations considered minor were: paresthesia without objective findings, clumsiness without objective findings, persistent headache, pseudopapilledema, benign intracranial hypertension, reactive depression, mood swings, cognitive disorders, severe anxiety, and behavioral problems (24). The diagnosis of NPSLE was based on the presence of one major criterion; or one minor criterion plus abnormality shown on electroencephalogram, brain scan, CSF examination or cerebral angiogram (24). SLE patients with NP manifestations were further classified in focal or diffuse manifestations, depending of the extend of the brain area involved (24) (Table 1).

In 1987, a consensus conference was held with the objective of ascertaining the level of agreement on NPSLE manifestations amongst a group of international experts with interest in NPSLE (25). The majority of participants considered the ACR criteria for NPSLE insufficient for use in clinical practice (25). In an exercise, starting with a list of 52 potential clinical, laboratory, and imaging manifestations of NPSLE, the first five ranked items [atypical psychosis, seizures, transverse myelitis, and global cognitive dysfunction (dementia)] were selected as the basis for further studies and possible expansion of the ACR classification criteria for SLE (25). Although a list of descriptors and elements important to diagnosis were provided, other researchers did not use this classification and subsequent validation studies were not performed (11).

Other classification criteria have been suggested based on the pathogenic mechanism and diagnostic tests (26, 27). These classifications divided central nervous system (CNS) manifestations into focal and diffuse. Focal manifestations included seizures, cranial neuropathies, cerebrovascular accidents and transverse myelopathy; whereas, diffuse manifestations included acute confusional state, psychosis, affective disorders, seizures, and cognitive dysfunction. Movement disorders (chorea, athetosis, hemibalismus, cerebellar ataxia, parkinson-like), peripheral nervous system and miscellaneous (headache, aseptic meningitis, pseudotumor cerebri, multiple sclerosis-like, Myasthenia gravis) were classified in different categories (26, 27).

A major deficiency of all of these classifications of NPSLE has been the lack of definitions for individual manifestations, lack of standardization for investigation < and diagnosis and the absence of validation studies (11).

In 1999, the ACR research committee, composed by experts from a variety of subspecialties including rheumatology, neurology, immunology, psychiatry and neuropsychology produced a standard nomenclature and set of case definitions for NPSLE (28, 29). Nineteen NP syndromes (Tables 1, 2) were defined and diagnostic criteria, criteria for exclusion, and specific diagnostic tests (laboratory and imaging evaluation) were defined (28, 29). The new ACR nomenclature system was intended to expand the neuropsychiatric criteria of the ACR classification criteria for SLE (23, 28–30). A patient could be considered to have NPSLE, if they met the case definition for neuropsychiatric lupus and *in addition* met 3 or more of the ACR (non-NPSLE) criteria for SLE (28, 29). However, the ACR classification criteria were not elaborated to replace clinical judgment or intended to make a clinical diagnosis in a given patient (28, 29).

**Table 2:** Central nervous system manifestation following ACR case definitions.

**Central nervous system manifestations**	**Peripheral nervous system manifestations**
Aseptic meningitis	Acute Inflammatory Demyelinating Polyradiculoneuropathy
Acute Confusional State	Autonomic Disorders
Anxiety Disorder	Cranial Neuropathy
Cerebrovascular Disease	Mononeuropathy
Cognitive Dysfunction	Myasthenia Gravis
Demyelinating Syndrome	Plexopathy
Headache	Polyneuropathy
Movement Disorder	
Mood Disorders	
Myelopathy	
Psychosis	
Seizures	

*Adapted from ACR Ad hoc Committee on Neuropsychiatric Lupus Nomenclature (28, 29)*.

In 2012, the Systemic Lupus International Collaborating Clinics (SLICC) revised and validated the American College of Rheumatology (ACR) SLE classification criteria in order to improve clinical relevance, meet stringent methodology requirements, and incorporate new knowledge in SLE immunology (31). The neurological domains were expanded to include seizures, psychosis, mononeuritis multiplex (in the absence of other known causes such as primary vasculitis), myelitis, peripheral, or cranial neuropathy (in the absence of other known causes such as primary vasculitis, infection, and diabetes mellitus), acute confusional state (in the absence of other causes, including toxic-metabolic, uremia, drug) (31).

## Studies applying ACR criteria

The ACR nomenclature and case definitions for NPSLE have been validated in a cross-sectional, population-based study (32). Forty-six SLE patients were compared to 46 age-, sex- and education matched individuals randomly selected from the Finnish population. NP manifestations were identified in 91% of SLE patients compared to 54% of controls. This provided a specificity of 46%. Due to the high prevalence of NP disease in both SLE and controls, the authors suggested exclusion of headache, anxiety, mild depression, mild cognitive impairment, and polyneuropathy without electrophysiological confirmation. With these modifications, the prevalence of NP manifestations fell to 46% in SLE and to 7% in controls, increasing specificity to 93% (32). These findings should to be taken into account in a future revision of the criteria.

Several other cohorts have applied the 1999 ACR diagnostic criteria for NPSLE and several meta-analysis have summarized the findings (33–45). Prevalence of clinical syndromes have been analyzed in a systematic review published in 2011, included 17 single-center articles with a total of 5057 SLE patients. NP manifestations were observed in 1439 (28.5%) SLE patients, ranging from 2.2 to 94.7% (37). Retrospective chart review studies had significant lower prevalence of NPSLE when compared to prospective studies (37). In decreasing order, the following CNS manifestations were reported: headache (12.2%), mood disorders (7.4%), seizures (7.0%), cognitive dysfunction (6.6%), and cerebrovascular disease (5.0%) (33). Other syndromes occurred in less than 5% of the cohorts (37).

Headache has been extensively studied in SLE patients. In a systematic review and the researchers concluded that the prevalence of all headache types, particularly that of tension-type headache, and migraine, does not differ between SLE patients and the general population (38), supporting the validation cohort results (31). However, headaches are associated with greater disease damage, higher disability and reduced cerebral gray matter volume when compared to SLE patients without headache (39–41).

Cognitive dysfunction was defined by the ACR as significant deficits in at least one of the following cognitive domains: simple or complex attention, reasoning, executive skills, memory, visual-spatial processing, language, and psychomotor speed, evaluated by standard 1-h battery of neuropsychologic tests (28, 29). The reliability and validity of this battery were later tested and established in native English speaking population (42). However, there are incomplete normative ethnicity, language and sex matched data, which leads to difficulties in the interpretation in certain populations and comparisons between cohorts (10). In addition, not all studies included in the meta-analysis have done the complete cognitive evaluation (37). When proper neuropsychologic testing was conducted in unselected SLE patients, the prevalence of cognitive dysfunction reported was significant higher (23–60%) (37, 43).

The association of autoantibodies and clinical syndromes have been analyzed in two independent meta-analysis (44, 45). In total, 17 studies with data on anti-NR2A/B antibodies in 2,212 SLE patients, 99 patients with other autoimmune diseases (e.g., antiphospholipid syndrome, myasthenia gravis, and autoimmune polyendocrine syndrome I) and 538 healthy controls were included. Overall pooled prevalence of serum/plasma anti-NR2A/B antibodies was higher in SLE patients [24.6% (95% CI 18.5–32.0%)] compared to other autoimmune diseases [14.8% (95% CI 2.2-56.9)] and healthy controls [7.6% (95% CI 4.6–12.4%)] (*p* = 0.001) (40). SLE patients with NP syndromes had more frequently NR2A/B antibodies [pooled OR = 1.607 (95% CI 1.041–2.479), *p* = 0.032] as compared to SLE without NP syndromes. Among the 19 NP syndromes, serum/plasma anti-NR2A/B antibodies were not specifically associated with any NP syndrome (44).

A second a meta-analysis included 41 articles to determine serum and cerebrospinal fluid (CSF) autoantibodies in patients with NPSLE and SLE (41). There was a significantly greater proportion of NPSLE patients with positive serum anti-cardiolipin antibodies (aCL) (OR = 1.63, 29 *p* = 0.016), lupus anticoagulants (LA) (OR = 1.91 *p* = 0.01), antiphospholipid antibodies (APL) (OR = 2.08, 30 *p* = 0.001), anti-ribosomal P antibodies (OR = 2.29, *p* < 0.001), anti-neuronal antibodies (OR = 9.50, *p* < 0.001) as compared to SLE patients without NP symptoms. In NPSLE patients, there was a significant increased prevalence of positive titers for CSF anti-neuronal antibodies (OR = 36.84, *p* = 0.001) as compared to SLE patients. Among the 19 neuropsychiatric syndromes, the presence of these serum autoantibodies were found to be associated with mood disorder, psychosis, cerebrovascular disease, seizure disorders, acute confusional state, cognitive dysfunction, headache, movement disorder, demyelinating syndrome, and polyneuropathy (45).

## Definition of NPSLE: does the ACR nomenclature still hold?

The guidelines to define neuropsychiatric nomenclature by the ACR in 1999 was the first step toward a uniform diagnostic criteria. It was an important contribution to the understanding of NPSLE; has been validated in independent cohorts and has been used by nearly all researchers since its publication, allowing epidemiological studies, comparisons between different cohorts and multicenter studies. Comparing the studies, the overall prevalence of NP manifestations is still variable, between 30 and 95%, and did not change when compared to studies done prior to 1999 (10, 11, 33). This variability can be due to study design, ethnicity, disease related aspects, referral bias, and the retrospective nature of many studies.

When considering individual patient care an important question to be answered is if the nature of the NP manifestations is vascular or inflammatory (8). Therefore, a few centers have proposed additional steps toward a more comprehensive classification system to support clinical judgment and improve translational research (46–53). In this setting, it is important to include in addition to definitions, variables such as temporal relationship of NPSLE to SLE diagnosis, presence of disease activity, presence of autoantibodies, as well as favoring, and confounding factors. Immunosuppressant can benefit SLE patients with inflammatory NP manifestations, with significant improvement of quality of life, whereas, SLE patients with non-related SLE manifestations may benefit primary from symptomatic treatment (50). In clinical practice, NP events presenting in SLE are too often attributed to an immune-mediated origin (51). In addition, the role of autoantibodies, cytokines and MRI findings in NP SLE can be better elucidated in the presence of a more comprehensive attribution model.

## Conclusion

In conclusion, the 1999 ACR case definition was an important step toward unifying definitions of important NP syndrome; and they have been used widely in epidemiological studies. However, to support clinical judgment and to improve patient care a more comprehensive model is needed, including additional clinical information. Attribution models, based on the ACR case definitions, may be the next step, to support translational research and clinical trials in NP manifestations.

## Author contributions

JFV, JCA, PRJ, RJO, and SA have made significant contribution to design, literature review, manuscript writing, and approved the final version.

### Conflict of interest statement

The authors declare that the research was conducted in the absence of any commercial or financial relationships that could be construed as a potential conflict of interest.
